# Middle ear pathologies in adults within the mining industry: A systematic review

**DOI:** 10.4102/sajcd.v67i2.679

**Published:** 2020-03-30

**Authors:** Ben Sebothoma

**Affiliations:** 1 Department of Speech Pathology and Audiology, Faculty of Humanities, University of the Witwatersrand, Johannesburg, South Africa

**Keywords:** Middle ear pathologies, Mining industries, Systematic review, Adults, Trends

## Abstract

**Background:**

Literature suggests that risk factors for middle ear pathologies, such as traumatic injuries and human immunodeficiency virus (HIV), exist in mines. However, studies on hearing health in mines seem to focus primarily on occupational noise-induced hearing loss and ignore middle ear pathologies. As a result, there is little documented evidence on the trends of middle ear pathologies in mine workers.

**Objectives:**

The aim of this study was to explore and document published evidence reflecting trends in middle ear pathologies in mine workers.

**Method:**

A systematic literature review of studies that reported middle ear pathologies in mine workers was conducted. Medline, CINAHL, PubMed, PsychInfo and Google Scholar databases were searched for studies in English published between January 1994 and December 2018 and reporting on trends in middle ear pathologies in mine workers.

**Results:**

Two research studies met the selection criteria and were included for analysis. One research study used tympanometry with 226 Hz probe tone, while another study used interviews to determine the presence of middle ear pathologies. While these studies indicate that middle ear pathologies exist in individuals working in mines, the evidence is limited.

**Conclusion:**

While current data indicate that individuals working in mines may present with middle ear pathologies of varying severities, the evidence is too small to provide a clear trend of middle ear pathologies in individuals working in mines. Therefore, the current limited data suggest a need for further studies to examine middle ear pathologies in individuals working in mines.

## Introduction

Although the mining industry plays an important role in the economic growth of any country, especially African countries, health hazardous conditions such as the auditory pathologies continue to be the biggest threats to this growth (Cawood, 2011). Consequently, the Mine Health and Safety Council (MHSC) of South Africa in 2003, which was revised in 2014, called for urgent action to prevent and manage auditory pathologies. However, the occupational noise-induced hearing loss (ONIHL) became the central focus in the literature, with little or no focus on middle ear pathologies (Amedofu, 2002; Chadambuka, Mususa, & Muteti, 2013; Kanji, Khoza-Shanagse, & Ntlhakana, 2019; Moroe & Khoza-Shangase, 2018; Musiba, 2015).

According to the World Health Organization (WHO), middle ear pathologies are amongst the most common auditory pathologies affecting over 700 million people worldwide (WHO, 2018). Although middle ear pathologies can be treated with simple antibiotics (Vouloumanou et al., 2009), the prolongation of middle ear pathologies can cause complications. According to WHO (2018), over 30 million people with acute otitis media (AOM) will develop chronic suppurative otitis media (CSOM), of which approximately 50% will develop permanent hearing loss. Kolo, Salisu, Yaro and Nwaorgu (2012) also found an association between CSOM and sensorineural hearing loss (SNHL). In addition, middle ear pathologies have also been linked with auditory processing difficulties (Villa & Zachetta, 2014). and intracranial complications (Sharma, Jaiswal, Barnerjee, & Garg, 2015).

Given the trends of middle ear pathologies across the world, it is plausible that these pathologies also exist in mines. Furthermore, literature has reported that risk factors associated with middle ear pathologies exist in mines. Donoghue (2004) reviewed occupational health hazards in mining and found that human immunodeficiency virus (HIV) and minor traumatic injuries were key amongst the health hazards. Hermanus (2007) argued that the existence of HIV in mining can be attributed to the living and working conditions of the miners. The United States Agency for International Development (USAID) also reported that the conditions of the mines may contribute to the risky sexual activities engaged by mine workers as a coping mechanism (Martins-Fonteyn et al., 2017).

Numerous studies have found an association between HIV and middle ear pathologies (Ensink & Kuper, 2017; Matas, Angrisani, Magliaro, & Segurado, 2014; Obasineke, Amdi, Ibekwe, Ezeanolue, & Ogisi, 2014; Sebothoma & Khoza-Shangase, 2018; Tshifularo, Govender, & Monama, 2013; Vajpayee, Negi, & Kurapati, 2013). These studies suggest that HIV incapacitates the immune system, making it ineffective to fight the invading pathogens. Consequently, middle ear pathologies occur from the opportunistic infections (Vajpayee, Negi, & Kurapati, 2013). The prevalence of middle ear pathologies in individuals living with HIV can be as high as 60% (Matas et al., 2014).

On the contrary, MØller (2006) argued that traumatic injury to the ear can interrupt the ossicular chain, reduce the sound energy into the ear and cause conductive hearing loss (CHL). In a prospective analytical study conducted by Sogebi, Oyewole and Mabifah (2018), 47.8% of the 205 participants with various forms of ear trauma presented with tympanic membrane perforation. Of these, 20.8% of the ear trauma were categorised to be accidental or self-inflicting injuries. Arguably, these accidental injuries may relate to the one described by Donoghue (2004) in the review. While sound reduction appears to be an immediate consequence of impaired middle ear system (MØller, 2006), non-pulsatile tinnitus and vertigo are also found to be associated with traumatic injuries of the middle ear (Delrue et al., 2016). These conditions can have a significant impact on individuals’ day-to-day living and affect the quality of life (Anderson, Parbery-Clark, White-Schwoch, Drehobl, & Kraus, 2013; Holman, Drummond, Hughes, & Naylor, 2019).

In light of the risk factors for middle ear pathologies that are reported to exist in mines, and the common trends of middle ear pathologies, the present study aimed to explore and document, through a systematic review, published evidences reflecting trends in middle ear pathologies in mine workers.

## Methods

### Data sources and literature search

This systematic literature review was conducted in line with the Cochrane collaboration guidelines in conjunction with the Preferred Reporting Items for Systematic Reviews and Meta-Analysis (PRISMA) (Moher et al., 2009). A comprehensive search was conducted on articles that had reported on middle ear pathologies in mine workers. A computer-aided search of online journal databases, including Medline, CINAHL, PubMed, PsychInfo and Google Scholar, was undertaken. The following keywords were used: ‘middle ear pathologies’ OR ‘middle ear disorders’ OR ‘middle ear infection’ OR ‘ear infection’ OR ‘otitis media’ OR ‘conductive hearing loss’ OR ‘hearing loss’ OR ‘hearing impairment’ OR ‘ear trauma’ OR ‘ossicular disorder’ AND ‘work-related’ AND ‘mining’ OR mineworkers.

### Inclusion criteria

The search was conducted on articles published between January 1994 and December 2018. This time frame was similar to the one used in another systematic review on mining workers and ONIHL (Moroe, Khoza-Shangase, Kanji, & Ntlhakana, 2018). Studies were included in this review if they were published in peer-reviewed journals, included mine workers and documented their middle ear status. Furthermore, studies had to be written in English. Studies were also included even if they were not randomised controlled trial (RCT) studies (Higgins & Altman, 2011).

### Data extraction and synthesis

**FIGURE 1:**
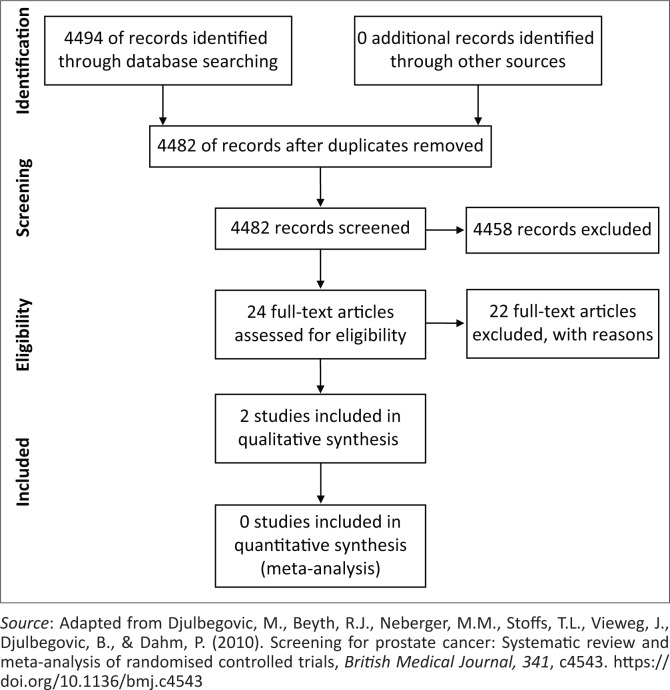
The Preferred Reporting Items for Systematic Reviews and Meta-Analysis flow diagram describing the inclusion of studies.

A total of 4494 titles were retrived. Of these, 12 studies were removed as these were duplicates. As a result, 4482 studies were considered and screened. Of the 4482, 4458 studies were excluded based on the titles and/or abstracts because they were not in line with the present study. A total of 24 articles were assessed for eligibility, but 22 studies were exlcuded with reasons (e.g. studies did not measure middle ear function). Finally, a total of 2 studies were included for analysis. The PRISMA flow diagram, showing the process of selecting studies for inclusion in this review is included (Figure 1).

### Ethical considerations

This article followed all ethical standards for a research without direct contact with human or animal subjects.

## Results

Table 1 summarises the results identified for the review through literature search. A total of 4494 studies were retrieved from all four databases. Of these studies, 4470 were excluded as they did not meet the selection criteria and 24 records were selected based on their titles and/or abstract. Of the 24 selected records, 22 were excluded because of duplication and the fact that they did not report on middle ear status of individuals working in mines. Finally, two research articles met the criteria for analysis. Table 2 summarises the studies included for analysis.

**TABLE 1:** Summary of research studies identified through database search.

Databases	Hits
Medline	1659
CINAHL	2176
PubMed	511
PsychInfo	145

**TABLE 2:** Middle ear pathologies in mine workers.

Study	Sample size	Middle ear measure	Findings	Mining industry
Landen et al. (2004)	*n* = 294	226 Hz tymp	6.4% presented with abnormal tympanometry	Sand and gravel mines
Saunders et al. (2013)	*n* = 59	Interview	14% reported prior ear drainage	Gold mine

### Measure of middle ear assessment

This review identified two research studies that had used different measures to determine the presence of middle ear pathologies in individuals working in mines. Landen, Wilkins, Stephenson & McWilliams, (2004) examined the middle ear status of the miners using tympanometry with 226 Hz probe tone. The cut-off values of tympanometry used in this study was adopted from the criteria described by Margolis and Shanks (1985). Saunders et al. (2013), on the contrary, used an interview method to determine whether participants presented with middle ear problems. Participants in this study were asked to indicate whether they have or had a history of ear discharge (otorrhoea).

### Rates of middle ear pathologies

One research study that used tympanometry with 226 Hz probe tone (Landen et al., 2004) reported that 6.4% (*n* = 19) of the participants recruited from the sand and gravel mine presented with some form of abnormal tympanometry. However, the types of abnormal tympanograms (e.g. type A) were not reported. These participants were ultimately excluded from the main study because the primary purpose of the study was to examine inner ear hearing loss. However, for the purpose of this review, this number (*n* = 19) was included for analysis. The study by Saunders et al. (2013), who used interview methods to determine the presence of middle ear pathologies, reported that 14% of the participants reported prior ear drainage (otorrhoea). In this study, only ear drainage was enquired about. Other middle ear-related symptoms, such as aural fullness, itchiness and otalgia, were not reported.

## Discussion

The aim of this systematic review was to summarise evidence reflecting trends in middle ear pathologies in individuals working in mines. Specifically, this systematic review aimed to look at the rates of middle ear pathologies, middle ear measures used, severity and risk factors. To the author’s knowledge, this is the first systematic review that explored and documented middle ear pathologies in individuals working in mines. Because of the heterogeneity of the studies included in this review, only qualitative synthesis was conducted.

This systematic review identified two research studies that reported on middle ear pathologies in individuals working in mines. These studies reported that middle ear pathologies exist (Landen et al., 2004; Saunders et al., 2013), perhaps in varying severities. For example, Saunders et al. (2013) reported that their study participants presented with otorrhoea. MØller (2006) indicated that otorrhoea is often consistent with advanced form of middle ear pathologies, such as chronic otitis media with effusion. However, because otoscope and tympanometry results were not reported, it is difficult to determine the nature of the middle ear pathologies.

Given that individuals working in mines are susceptible to traumatic injuries, and have been shown to have high prevalence of HIV (Hermanus, 2007), it is surprising and concerning that middle ear pathologies in this population are largely ignored. Baltazar et al. (2015) reported that the conditions of the mines may contribute to the risky sexual acitiviies engaged by mine workers. Tuan (2010) found that miners engage in risky sexual behaviours as a coping mechanism. Baltazar et al. (2015) also reported that 22.3% of Mozambicans working in South African mines were living with HIV. Of those, approximately 75% of the participants did not know their HIV status. This means that the majority of participants (75%) were not on HIV treatment, making them even more prone to middle ear pathologies (Obasineke et al., 2014; Van der Westhuizen, Swanepoel, Heinze, & Hofmeyr, 2013).

The findings of this systematic review suggest that hearing conservation programmes (HCPs) need to include middle ear assessment. This may provide a better management and quality of life of individuals working in mines. The author suspects partly that the paucity of evidence of this important auditory information may be because of lack of involvement of audiologists in mines, as highlighted by Moroe and Khoza-Shanagse (2018).

## Conclusion

This is the first systematic review to look at the trends of middle ear pathologies in adults working in mines. However, this review highlights the paucity of research in the area of middle ear pathologies in individuals working in mines. There were only two research studies that reported on middle ear pathologies in adults working in mines. While these studies indicate that middle ear pathologies exist in adults working in mines, evidence is too small and weak to suggest any trends. Therefore, the findings of this review suggest that there is a need for assessment of middle ear pathologies in individuals working in mines. The inclusion of middle ear assessment will contribute to early detection and intervention of middle ear pathologies, thus potentially increasing the quality of life.
